# Preference for ugly faces? —A cognitive study of attentional and memorial biases toward facial information among young females with facial dissatisfaction

**DOI:** 10.3389/fpsyg.2022.1024197

**Published:** 2022-11-02

**Authors:** Lan Zhu, Huan Zhou, Xiaogang Wang, Xiao Ma, Qiaolan Liu

**Affiliations:** ^1^West China School of Public Health and West China Fourth Hospital, Sichuan University, Chengdu, China; ^2^School of Education and Psychology, Southwest Minzu University, Chengdu, China

**Keywords:** young females, facial dissatisfaction, cognitive bias, behavioral experiments & ERP experiments, facial attractiveness

## Abstract

Dissatisfaction with facial appearance is one of the strongest contributors to body image disturbance among young Chinese females and leads to a series of psychological and behavioral disorders. By conducting behavioral and ERP experiments, this study illustrates how young females in China with facial dissatisfaction process different levels of facial attractiveness. Experiments 1 and 2 are behavioral experiments in which the dot-probe paradigm was used to explore the participant’s attentional bias to facial attractiveness. The results showed that regardless of whether the face image was presented above or below the threshold, young females with facial dissatisfaction exhibited attentional orientation toward lowly attractive faces and attentional avoidance to both lowly and highly attractive faces, while the control group showed difficulty in attentional disengagement from highly attractive faces. In experiment 3, the learning-recognition task was used to examine mnemonic bias toward facial attractiveness among females with facial dissatisfaction, and EEG data were also recorded during the encoding and retrieval phases. The study found that young females with facial dissatisfaction exhibited a mnemonic preference for lowly attractive images at both the encoding and retrieving stages, with higher P1, N170, P2, and N300 induced by lowly attractive faces, while the control group preferred highly attractive faces. In conclusion, young females with facial dissatisfaction tend to exhibit attentional orientation and mnemonic bias toward lowly attractive faces.

## Introduction

Individuals with facial attractiveness have more advantages in job hunting, mate selection, and interpersonal communication. Facial dissatisfaction, compared with other image dimensions, can more easily develop into body image disorder, which is defined as negative cognition, negative emotional experience, and corresponding behavior regulation related to one’s own appearance (Jackson and Chen, 2008a). In other words, facial dissatisfaction can be considered the epitome of body image disturbance to some extent. A large number of studies have found that individuals with body image disturbance have a cognitive bias toward body-related information (Mitchison and Mond, 2015; Aktar et al., 2019), suggesting that individuals with body image disturbance will selectively pay attention to (Maner et al., 2006; Shafran et al., 2008; Nijs et al., 2010; Veenstra and de Jong, 2012; Folkvord et al., 2015; Cass et al., 2020), evaluate (Levine and Piran, 2004; Chen and Jackson, 2005; Chen et al., 2006; Jackson and Chen, 2008a; Glashouwer et al., 2016), and remember negative information consistent with negative body images, such as obesity information (Williamson et al., 2000; Smeets et al., 2011; Brockmeyer et al., 2018; von Spreckelsen et al., 2018). Additionally, they resist positive information that is inconsistent with negative body images such as slim figures (Rieger et al., 1998; Dobson and Dozois, 2004; Smeets et al., 2008). Research of this type mostly focuses on the problem of body image disturbance from the perspectives of body and weight, while research on the dimension of facial appearance is rather rare. Compared with young American females, who pay more attention to body shape, Asian females, especially Chinese young females, believe that beautiful faces are more important (Chen and Jackson, 2005; Chen et al., 2006; Hua, 2009; Luo, 2013). Highly attractive (HA) pictures are considered to be more advantageous for health (Shackelford and Larsen, 1999; Henderson and Anglin, 2003; Little et al., 2011), spouse selection (McNulty et al., 2008; Ma-Kellams et al., 2017; Arnocky, 2018), career trajectory (Halford and Hsu, 2020), and decision-making (Kowner, 1996; Chen et al., 2012; Atari et al., 2017; Kramer et al., 2020). Chen and colleagues’ research on physical dissatisfaction among Chinese adolescents found that facial appearance is one of the main sources of negative body images among Chinese female adolescents, and dissatisfaction with facial appearance is one of the most important predictors of negative physical self-image among young females. A negative physical self-image can predict eating disorders (Chen and Jackson, 2005, 2006; Chen et al., 2007; Jackson and Chen, 2008a), and is highly related to a series of psychological and behavioral disorders, such as anxiety, depression, blind dieting, and plastic surgery, as well as diseases, including diet disorders and anorexia nervosa (Chen and Jackson, 2006; Verplanken and Velsvik, 2008; Jackson and Chen, 2008b; Pawijit et al., 2019). Therefore, studying the cognitive processing features of facial information in young females with facial dissatisfaction is the basis for understanding, preventing, and correcting their potential psychological and behavioral problems.

Most cognitive neuroscience and clinical psychology studies have found that threatening stimuli tend to capture individuals’ visual attention (Massar et al., 2011; Cho and Lee, 2013; Richards et al., 2014; Gupta et al., 2019). In recent years, many studies have found that individuals have attentional biases toward different types of threat stimuli after classification (Rinck and Becker, 2006; Shafran et al., 2008; Vrijsen et al., 2009). For example, Shafran et al. (2008) employed the traditional dot-probe paradigm to study the attentional bias of patients with eating disorders toward food, bodies, and other stimuli. Patients with eating disorders responded faster to the probe when it was in the same location as the negative body-shape stimuli (consistent condition) than when it appeared in the opposite location (inconsistent condition), indicating attentional vigilance to the negative body-shape stimuli. Related studies have revealed components of attentional bias by comparing the differences in response time, which functioned as the reflection of attention vigilance and attentional avoidance, between the two distinctive conditions (Wade and Lowes, 2002; Koster et al., 2005; Dong et al., 2017). During the latter part of the experiment, the researchers modified the dot-probe experimental paradigm, adding neutral stimulus pairs (neutral conditions), and distinguished facilitation and difficulty in attentional disengagement by comparing the difference in response time between inconsistent conditions and neutral conditions. For instance, a study on the attentional bias of patients with anxiety found that participants with anxiety had difficulty in attentional disengagement when confronted with threatening information (Koster et al., 2004; Rinck and Becker, 2006; Smeets et al., 2008). Based on this paradigm, researchers controlled the stimulus presentation time at approximately 20 ms and accompanied by front−/back-masking, which can examine the features of attentional processing below the threshold of consciousness. For example, Mogg and his colleagues employed the visual dot-probe with masking to examine the subliminal attention processing of negative faces in highly anxious participants and found that highly anxious participants showed attentional vigilance for threat faces relative to neutral faces, while lowly anxious participants showed attentional vigilance for happy faces relative to neutral faces (Mogg and Bradley, 1999; Mogg et al., 2005). Tobacco-deprived smokers had attentional vigilance to subliminal tobacco-related stimuli (Leventhal, 2010; Leventhal et al., 2010). Therefore, studies have compared the specific components of attentional bias to explain the cognitive processing mechanism of special information by illustrating the source and performance of this bias. However, a large number of study results proved that attention bias is not consistent, thereby indicating the need for further clarification. In addition, the cognitive bias of individuals with body image disturbance toward body information is also manifested in the selective memory for negative body information in the retrieving stage. For example, patients with eating disorders and obesity were able to recall more food and weight-related information in a study comparing their difference in the amount of recall of food and weight-related information (Ogden et al., 2017). Researchers used the word classification judgment-retrieving task to investigate the memory features of body words in Chinese and American young females with fat group and found that compared with females in the control group, the fat group forged fat as a more negative impression on fat-body words and was better in remembering these words (Chen and Jackson, 2005). Some researchers have also examined the attentional bias of face dissatisfied women to face-related stimuli based on the dot-probe paradigm combined with eye tracking technology (Kou et al., 2016). It revealed that women with facial dissatisfaction had attentional orientation and maintenance, at least initially, toward unattractive faces but showed overall attention maintenance to attractive ones. Using facial words as experimental materials, researchers found that women who were not satisfied with their facial attractiveness showed initially attentional orientation and maintenance to negative faced-related words, and also showed a speeded attention orientation to positive face-related words (Kou et al., 2015). However, few studies have combined attentional and memorial processes to explore the processing mechanism of different types of face-related stimuli among individuals with body dissatisfaction. Considering the limited research of this type and the information processing mechanism remaining unclear, refinement and perfection are urgently needed.

Some studies have focused on the neural basis of cognitive processing of negative body images, merely focusing on brain imaging research of food information for individuals with eating disorders. For instance, a study on word processing among patients with anorexia nervosa by using food and body words as experimental materials found that when the word ‘Thin’ appears, the medial frontal cortex, insular lobe, temporal lobe, occipital area, and frontal lobe of patients with anorexia nervosa are rather active; when the word ‘Fat’ appears, the left prefrontal cortex, middle frontal gyrus and superior parietal lobule of normal participants are significantly activated (Redgrave et al., 2008). After being exposed to more detailed negative body features, it was found that the left amygdala and parahippocampal gyrus were more activated (Jastreboff et al., 2013; Carnell et al., 2014). Researchers believe that activation of the amygdala is related to the fear caused by negative body words and the detection of threat information in the environment (Uher et al., 2004; Yokum et al., 2011; Spangler and Allen, 2012; Sweet et al., 2012; Frank et al., 2013; Jastreboff et al., 2013; Carnell et al., 2014; Sweitzer et al., 2018). The left prefrontal cortex is related to emotion processing, involving attention, evaluation, judgment, and adjustments of emotional stimuli. Event-related potential (ERP) research in this area is mostly used on clinical participants, involving changes or impairment of cognitive function and investigating changes in P300 (Otagaki et al., 1997; Dodin and Nandrino, 2003; Hachl et al., 2003). However, for nonclinical participants, P300 is not sensitive enough. A difference in both N2 and P2 instead of P300 was detected in the research conducted by Yi et al. (2008). They found that, compared with normal pictures, fat body pictures induced greater N2 and P2 in the group with weight dissatisfaction. The researchers believe that P2 and N2 reflect the spatial attentional bias at the early stage and function as the primary processing of visual stimuli. It shows that in the early stage of attention processing, negative body image will affect the allocation of attentional resources, resulting in attention processing bias toward negative body-related information. Compared with clinical participants, nonclinical participants may exhibit an earlier attentional bias toward negative body-related information.

During face processing, attractive faces can capture attention and make it difficult to disengage (Maner et al., 2007; Sui and Liu, 2009; Ma et al., 2019). Studies have shown that when faces are presented, participants will quickly direct their attention to the attractive face area instead of the unattractive one, indicating a faster attention orientation towards the attractive face area (Leder et al., 2010, 2016; Mitrovic et al., 2016). A large number of ERP studies have found that faces can induce early ERP components even in the early processing stage, such as P1, N1, and N170, and these early components are relatively stable for face processing (Ackles and Cook, 1998; Hillyard et al., 1998). P1 is commonly considered to be related to the processing of low-level physical attributes or the classification of stimuli. It is believed that P1 may reflect the early stage of face processing and perhaps the comprehensive representation and encoding of simple facial attributes, which was consistent with the idea of Itier and Taylor (2002, 2004). N170, a specific component of face processing, is mainly distributed in the temporal-occipital area of the brain (Bentin et al., 1996). It usually has the largest amplitude at electrodes such as P8 (T6) or PO8 or O2 and often has a right-hemisphere dominance, which represents the processing of face structure and the processing of the spatial relationship of face parts or expression (Rossignol et al., 2012, 2013; Tanaka, 2018). Mariz et al. found that HA faces induce stronger N170 on the occipital and temporal electrodes than lowly attractive (LA) and unattractive faces (Marzi and Viggiano, 2010). In conclusion, researchers have found features of dynamic changes in early and late ERP components induced by attractive faces. Whether young females with facial dissatisfaction process different-level facial attractiveness with bias and whether there are any differences in the preference for HA faces between young females with facial dissatisfaction and those without facial dissatisfaction are issues that need to be further explored.

This study adopts a modified dot-probe paradigm and a learning-recognition paradigm to explore the features of attention and memory processing of faces among young females with facial dissatisfaction by subliminally and supraliminally presenting HA, mediumly attractive (MA), and LA face pictures. We assume that females with facial dissatisfaction have a bias in attention and memory processing toward LA pictures. The LA faces evoke larger amplitudes of ERP components, which are not proven in the control group.

## Materials and methods

### Measurement and experimental materials

#### Measurement

The Facial Negative Physical Self Scale (NPSS-F) was used to categorize participants. The NPSS-F contains 11 items, and each item is rated on a 5-point Likert scale from *1 = not at all like me* to *5 = very much like me*. Individuals with an average score of 2.5 or more are defined as the facial dissatisfaction (FN) group (hereafter referred to as the FN group), and individuals with an average score of less than 1 are defined as the control group (Chen et al., 2006). The Cronbach alpha coefficient was 0.88 in this study.

#### Experimental materials

Female face images with neutral emotional expressions were collected from the open access database of Google that was processed uniformly by Photoshop and evaluated with unified standards. We collected 828 pictures of Chinese female faces (approximately 20–30 years of age) on the internet and used Photoshop to process the face pictures uniformly, balancing the differences in the physical attributes of each level of the pictures and removing the external features of the face such as hair, ears, and neck. All face pictures are grayscale with a size of 15 × 15 cm and pixels of 425 × 425. There are no familiar characters, such as the faces of celebrities. Then, the attractiveness, arousal, pleasantness, dominance, and emotional valence of the face pictures were evaluated. First, 10 female students were asked to initially classify the pictures into HA pictures, MA pictures, and LA pictures (just for now). A total of 628 pictures with the highest assessing consistency were selected for the next stage. Eighty female college students were asked to assess the facial attractiveness, emotional valence, arousal, pleasantness, and dominance of these faces. Then, 160 HA pictures, 160 MA pictures, and 160 LA faces were selected with neutral facial expressions, moderate dominance, and arousal. One-way ANOVA showed that there were significant differences in the attractiveness and pleasure scores between the HA (*M* = 7.77, SD = 0.79), MA (*M* = 4.54, SD = 0.73), and LA pictures (*M* = 1.99, SD = 0.80), *F*(2, 477) = 1125.12, *p* < 0.001; *F*(2, 477) = 859.89, *p* < 0.001. The differences in arousal and dominance between the three types of faces were not significant (*p* = 0.400; *p* = 0.347).

### Participants

Female undergraduates at a Chinese university were recruited to complete the NPSS-F and telephone interviews. Females with an average score of at least 2.5 on the NPSS-F were assigned to the FN group, and females with an average score of 1.0 or below on the NPSS-F were assigned to the control group. The exclusion criteria were as follows: chronic illness, mental illness, smoking and alcohol abuse in the last 2 years, and being normal visual acuity or corrected visual acuity right-handed.

In experiments 1 and 2, 80 participants were included in the FN group, with an average age of 18.32 ± 0.56 years old, and another 80 participants were included in the control group, with an average age of 19.17 ± 1.11 years old. The age difference between the two groups was not significant (*p* = 0.867), but the difference in the NPSS-F scores was significant (*F*(1, 158) = 18.342, *p* < 0.001). Participants in both groups were right-handed and were compensated after the experiment.

In experiment 3, 30 participants were included in the FN group, with an average age of 20.12 ± 0.44 years old, and another 30 participants were included in the control group, with an average age of 19.09 ± 1.21 years old. The age difference between the two groups was not significant (*p* = 0.657), but the difference in the NPSS-F scores was significant (*F*(1, 58) =28.35, *p* < 0.001). There was no history of brain injury or mental disorders in the two groups. After the experiment, the participants were awarded corresponding guerdon (see Table 1).

**Table 1:** Demographic data of experiments FN group and control group (*M* ± *SD*).

Experiment number	Grouping	*N*	Age	NPSS-F score
Experiment 1 and Experiment 2	FN group	80	18.32 ± 0.56	2.74 ± 0.47
	Control group	80	19.17 ± 1.11	0.77 ± 0.14
Experiment 3	FN group	30	20.12 ± 0.44	2.63 ± 0.75
	Control group	30	19.09 ± 1.21	0.78 ± 0.22

### Procedure

The research contains 3 experimental tasks. Experiments 1 and 2 used a modified dot-probe paradigm by presenting face stimuli subliminally and supraliminally to examine the attentional biases towards faces in young females with facial dissatisfaction. At the beginning of each trial in experiment 1, a white fixation point (+) was presented in the center of the black screen for 800–1,000 ms, followed by a blank screen for 100 ms, and then paired images (HA-neutral pictures, LA-neutral pictures, neutral-neutral pictures; hereinafter referred to as H-N, L-N, N-N) were displayed in the left and right boxes of the screen for 500 ms. One hundred milliseconds after the paired pictures disappeared, a black dot with a diameter of 0.3° was presented in the box on the left or right of the screen for 200 ms, and the participants were asked to judge where the black dot appeared. In experiment 2, the paired pictures were displayed for 20 ms, and then masking stimuli appeared for 100 ms to make sure that the paired pictures were presented subliminally, with the rest of the process the same as in experiment 1. In experiments 1 and 2, the participants were required to judge the position of the dot after it appeared, which would disappear after the participant responded, after which the experiment entered the next trial. In this experiment, if the dot appeared on the left side of the screen, the participant was asked to press the ‘Q’ key or the ‘P’ key if it was on the right. The experiment was divided into four blocks, and each block contained 60 pairs of paired pictures (HN, LN, NN every 20 pairs), for a total of 240 trials. Before the formal experiment, 12 trials were administered for practice. All participants rested for 2 to 3 min after each block.

**Figure 1:**
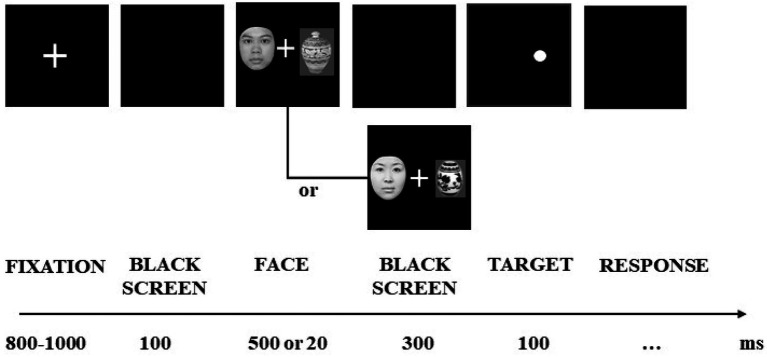
Sample of experimental procedure in experiment 1.

**Figure 2:**
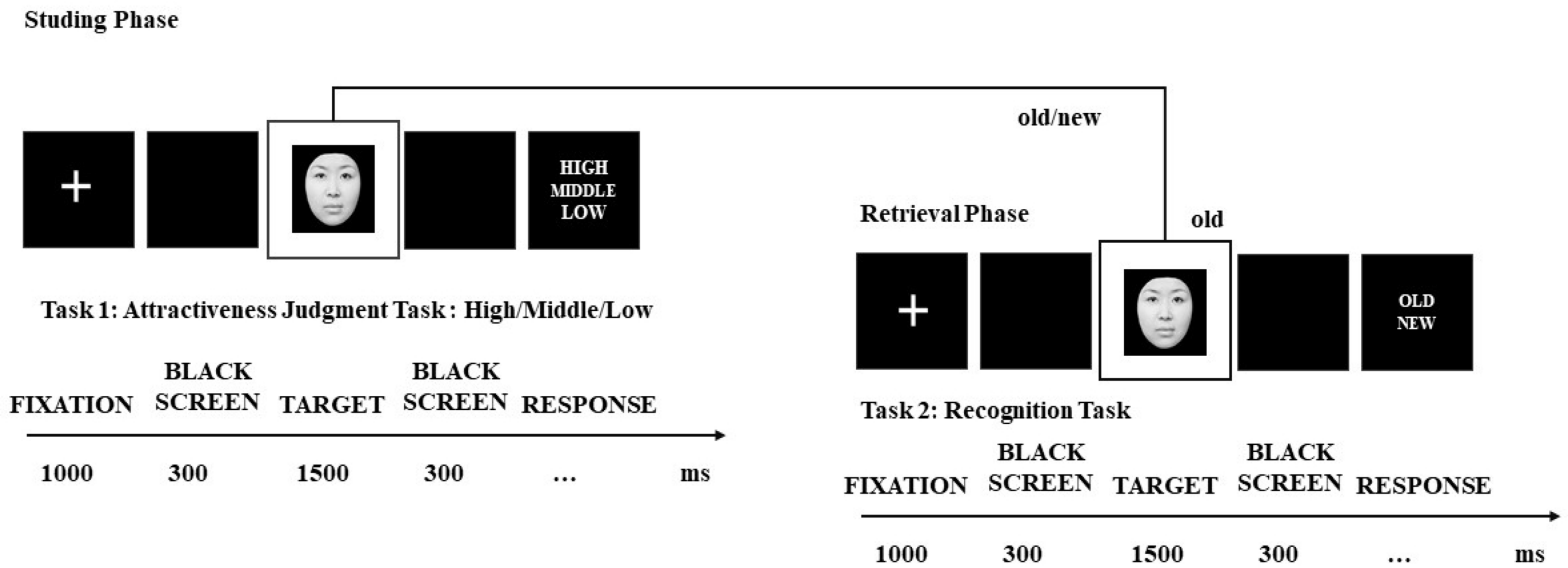
Sample of experimental procedure in experiment 3.

In experiment 3, the learning-recognition paradigm was employed to examine mnemonic bias toward facial attractiveness among females with facial dissatisfaction, and EEG data were also recorded during the encoding and retrieval phases. In the learning phase, at the beginning of each trial a white fixation point was presented in the center of the black screen for 1,000 ms, followed by a blank screen for 300 ms, and then followed by a face for 1,500 ms. Participants were required to complete the judging task of the attractiveness of faces. At last, a blank screen was presented for 500 ms, and then, the next trial began. In the learning phase, there were 240 pictures of female faces, including 80 HA pictures, 80 MA pictures, and 80 LA pictures. The learning phase had 2 blocks, and each block contained 120 face pictures, for a total of 240 trials. The participants rested for 2 to 3 min after each block. In the retrieving phase, the procedure was consistent with the learning phase, but participants were required to judge whether the face was old or new (Figure 1). The order in which the faces were presented was random to control the sequence effect. There were 480 female face pictures in the retrieving phase, including 160 HA pictures, 160 MA pictures, and 160 LA pictures, and we mixed half of the pictures that had been learned with those that had not been learned. The retrieving phase had four blocks, and each block contained 120 face pictures, for a total of 480 trials. The participants rested for 2 to 3 min after each block. Before the formal experiment, 12 practice trials were performed (see Figure 2).

### Devices and recording

Experiments 3 adopted recording and analysis systems developed by Brain Products (Germany) to record EEG in accordance with the International 10–20 system extension of 64 conductive pole caps. The referring electrodes were placed on the right and left mastoids, and the average value of the bilateral mastoids was used as a reference for offline analysis. The ground electrode was at the midpoint of the line connecting Fpz and Fz in the forehead. Horizontal electrooculography (HEOG) was recorded by placing electrodes approximately 1.5 cm outside both eyes, and vertical electrooculography (VEOG) was recorded by placing electrodes at the upper and lower sockets of the left eye. The scalp resistance at each electrode was kept below 5 kΩ. The filtered bandpass was 0.05 to 80 Hz, and the sampling frequency was 500 Hz/channel. After completing the continuous recording of the EEG, the data were processed offline, and electrooculography was automatically corrected by automatically eliminating wave amplitudes greater than ±80 μV as invalid records. The correct EEG responses were superposed by counting the participants’ responses according to the type of task, and the number of superpositions was greater than 60. The analysis window was fixed from −200 to 800 ms, with −200 to 0 ms serving as the baseline for correction.

### Data analysis

In experiments 1 and 2, 3 (Picture type: H-N, L-N, and N-N) × 2 (Consistency: the dot was presented in the same/different position of the target face) × 2 (Group: FN group and control group) repeated measure ANOVAs were conducted on the accuracy rate and response time of the two groups of participants.

In experiments 3, 2 (Group: FN group/control group) × 3 (Picture type: HA, MA, LA) repeated measure ANOVA was conducted on the accuracy rate and the reaction time of the two groups of participants during retrieving phase. ERP data may include the peak value and latency of ERP components during the learning and retrieving phases. According to the average EEG chart and EEG brain map, electrode points were used to analyze P1 (100 ~ 150), N170 (150 ~ 200), P2 (200 ~ 250), and N300 (250 ~ 300): P1 (PO7, PO8, O1, O2, Oz); N170 (PO7, PO8, P7, P8, O1, O2, Oz); P2 (CP5, CP3, CP1, P5, P3, P1, PO3, POz); and N300 (TP7, TP8, PO7, PO8, P7, P8, O1, O2, Oz). 2 (Group: FN group/control group) × 3 (Picture type: HA, LA MA) × 2 (Electrode position: left and right) repeated measure ANOVAs were conducted on the peak and latency of the above components. When analyzing the left and right effects, the midline electrode points were not included. The *p* value in the variance analysis was adjusted by the Greenhouse Geisser correction, and the pairwise comparison was corrected by the Bonferroni correction. The EEG brain maps were derived from 64-lead data.

## Results

### Behavioral results

The 3 × 2 × 2 repeated measure ANOVAs for the accuracy rate in experiments 1 and 2 did not reveal any significant main effects and interaction effects (Ps > 0.05). The participants involved in the behavioral experiments all had a correct rate of at least 95%, so all participants were retained. When there were participants with 4–11 errors (*M* = 7.4, SD = 2.86), only correct responses were collected (excluding response times data beyond plus or minus three standard deviations).

#### Attentional orientation

The 2 (Group: FN group and control group) × 3 (Experimental conditions: H-N, L-N, N–N) repeated measurement ANOVAs were conducted on the reaction time under the consistent condition in experiments 1 and 2, respectively. It revealed a significant interaction in experiments 1 and 2 (*F*(2, 316) = 12.898, *p* < 0.001; *F*(2, 316) = 15.425, *p* < 0.001). Simple effect analysis found that the response time of the L-N pictures in the FN group was significantly shorter than that of the N–N pictures in experiments 1 and 2 (*F*(1,158) = 11.152, *p* = 0.028 and *F*(1,158) = 11.807, *p* = 0.017, respectively), but there was no significant difference in response time between the three conditions in the control groups (Ps > 0.05), indicating that the FN group showed an attentional orientation to LA faces. All main effects were not significant (Ps > 0.05).

#### Attentional avoidance

The 2 (Group: FN group, control group) × 3 (Experimental conditions: H-N, L-N, N–N) repeated measurement ANOVAs were conducted on the reaction time under the consistent condition in experiments 1 and 2, respectively. The Group × Experimental conditions had a significant interaction effect in experiments 1 and 2 (*F*(2, 316) =3.059, *p* = 0.047; *F*(2, 316) =12.099, *p* < 0.001). Simple effect analysis found that reaction times in the H-N consistent condition of the FN group were significantly longer than that in the N–N condition (*F*(1,158) =33.292, *p* < 0.001; *F*(1,158) =33.292, *p* < 0.001), but there was no significant difference in response time between the three conditions in the control group (Ps > 0.05), indicating that the FN group showed an attentional avoidance to HA faces. All main effects were not significant (Ps > 0.05).

#### Difficulty in attentional disengagement

The 2 (Group: FN group, control group) × 3 (Experimental conditions: H-N, L-N, N–N) repeated measurement ANOVAs were conducted on the reaction time under the inconsistent condition in experiments 1 and 2, respectively. It revealed that the interaction effect was significant (*F*(2, 316) =3.059, *p* = 0.047; *F*(2, 316) =12.099, *p* < 0.001). Simple effect analysis found that there was no significant difference in response time between the three conditions in the FN group (Ps > 0.05), but reaction times in the H-N inconsistent condition of the control group were significantly longer than that in the N–N condition (*F*(1, 158) =9.458, *p* = 0.002; *F*(1, 158) = 6.425, *p* = 0.011), indicating that the control group had difficulty in attentional disengagement from HA faces. All main effects were not significant (Ps > 0.05).

#### Facilitation in attentional disengagement

In experiments 1 and 2, the 2 (Group: FN group, control group) × 3 (Experimental conditions: H-N, L-N, N–N) repeated measurement ANOVAs for the response times under the inconsistent condition were conducted. There were significant interaction effects (*F*(2, 316) =3.059, *p* = 0.047; *F*(2, 316) =12.099, *p* < 0.001). In the FN group, reaction times in the L-N consistent condition were significantly shorter than that in the N–N condition (*F*(1, 158) =33.292, *p* < 0.001; *F*(1, 158) =33.292, *p* < 0.001), but there was no significant difference in response time between the three conditions in the control group (Ps > 0.05), indicating that the FN group showed facilitation in attentional disengagement from LA faces. All main effects were not significant (Ps > 0.05).

#### Attractiveness judgment in the encoding phase

In experiment 3, the classification results of the participants and the classification criteria of HA, MA, and LA faces obtained by standardizing the face materials were analyzed by repeated measures ANOVA. The results showed that the difference between the participants’ classification criteria for the three types of faces in the pre-experiment and the correct judgment rate was not significant (Ps > 0.05), indicating that the participants’ judgments are highly consistent with the standardized results, according to which the statistics of correct rate and response time of the participants toward the three types of faces were collected. There was no significant difference between the two groups in the accuracy of the judgment of facial attractiveness (Ps > 0.05). There was an interaction effect of Group × Picture type in response time (*F*(2, 58) = 6.214, *p* = 0.006), and the FN group responded significantly slower to HA pictures relative to MA pictures and LA pictures (*p* = 0.04, *p* = 0.01). The difference in reaction time between the MA pictures and LA pictures was not significant (*p* = 0.08). The difference in the reaction time of the control group to the three types of attractive faces was not significant (Ps > 0.05). All main effects were not significant (*p* = 0.109; *p* = 0.242).

#### Mnemonic bias in the retrieval phase

The 2 (Group: FN group/control group) × 3 (Picture type: HA, MA, LA) repeated measurement ANOVAs on accuracy and reaction time did not reveal any significant main effects (Ps > 0.05), but the interaction effect was significant (*F*(2, 58) =5.69, *p* = 0.016; *F*(2, 58) =98.091, *p* < 0.001). The accuracy and reaction time between the picture types in the FN group were significantly different (*F*(2, 58) =5.67, *p* = 0.024; *F*(2, 58) =4.18, *p* = 0.05), and they exhibited the highest accuracy rate and the fastest response for LA pictures, while there was no significant difference between HA pictures and MA pictures (Ps > 0.05), indicating the FN group showed mnemonic bias towards LA faces. The control group had no significant difference in the accuracy rate and response time between the three types of pictures (Ps > 0.05).

### ERP results

**Figure 3:**
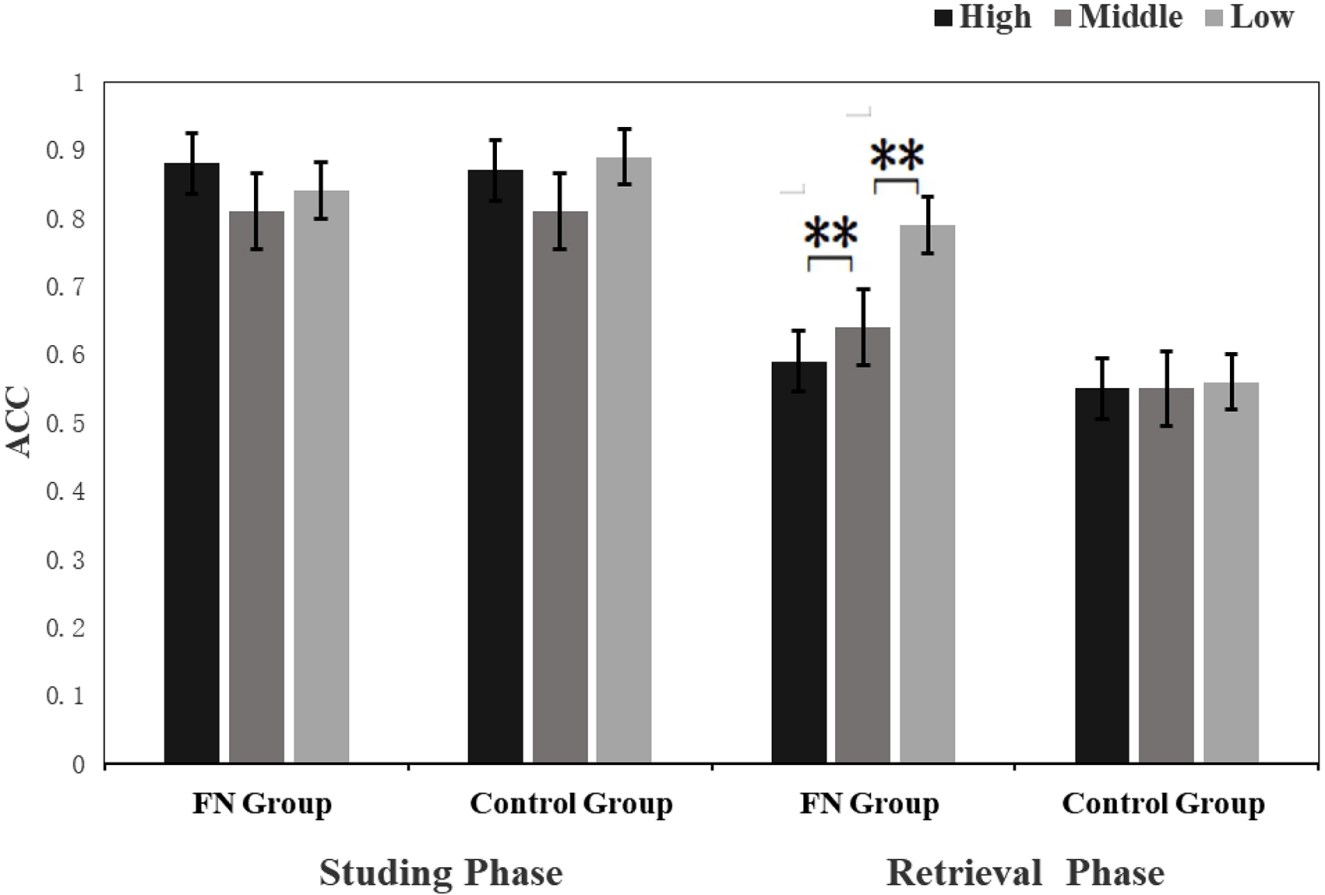
Percentage of correct accuracy (ACC) to attractive faces in the learning phase and retrieval phase. **p < 0.05.

**Figure 4:**
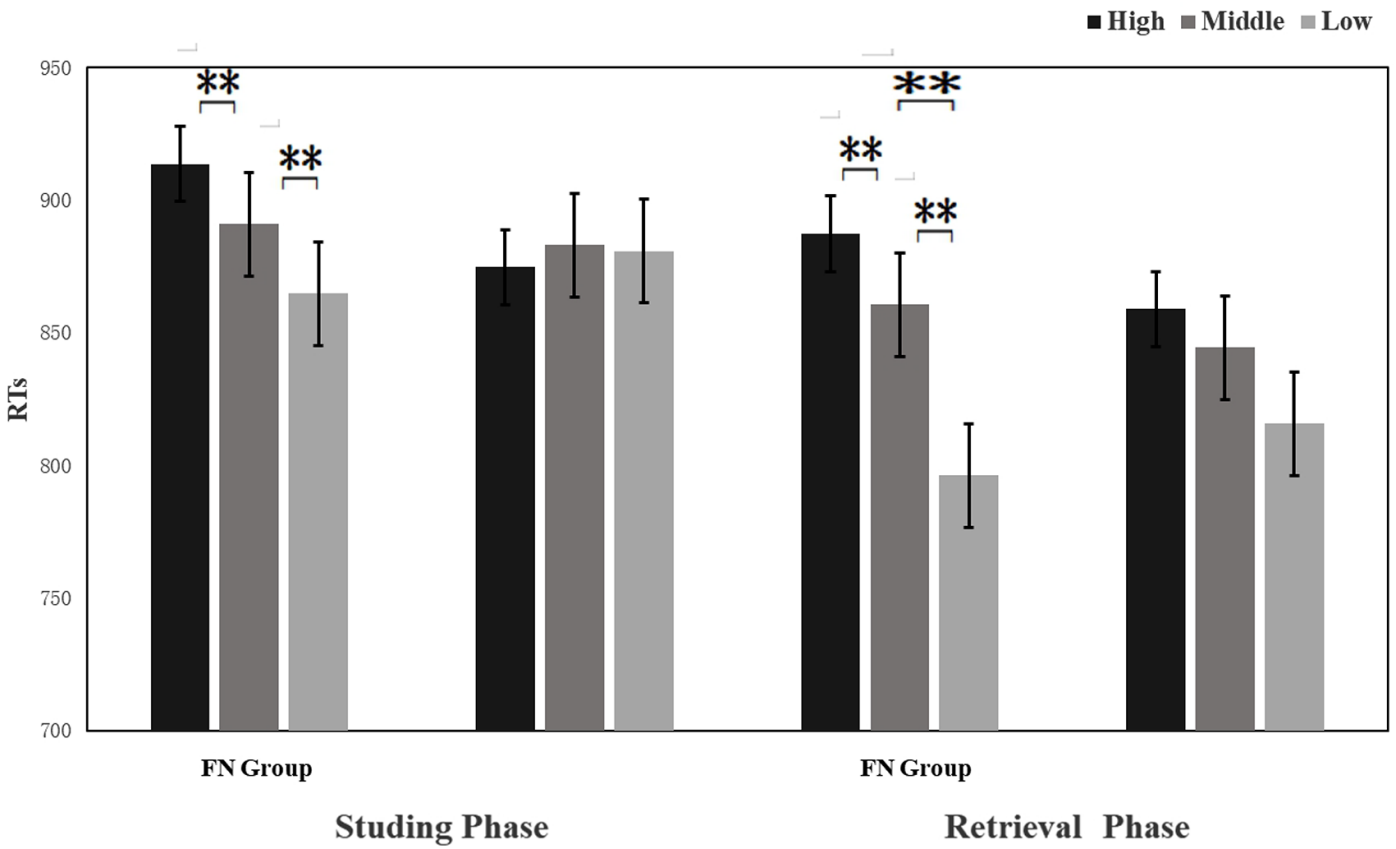
Reaction times (RTs) in response to attractive faces in the learning phase and retrieval phase. **p < 0.05.

**Figure 5:**
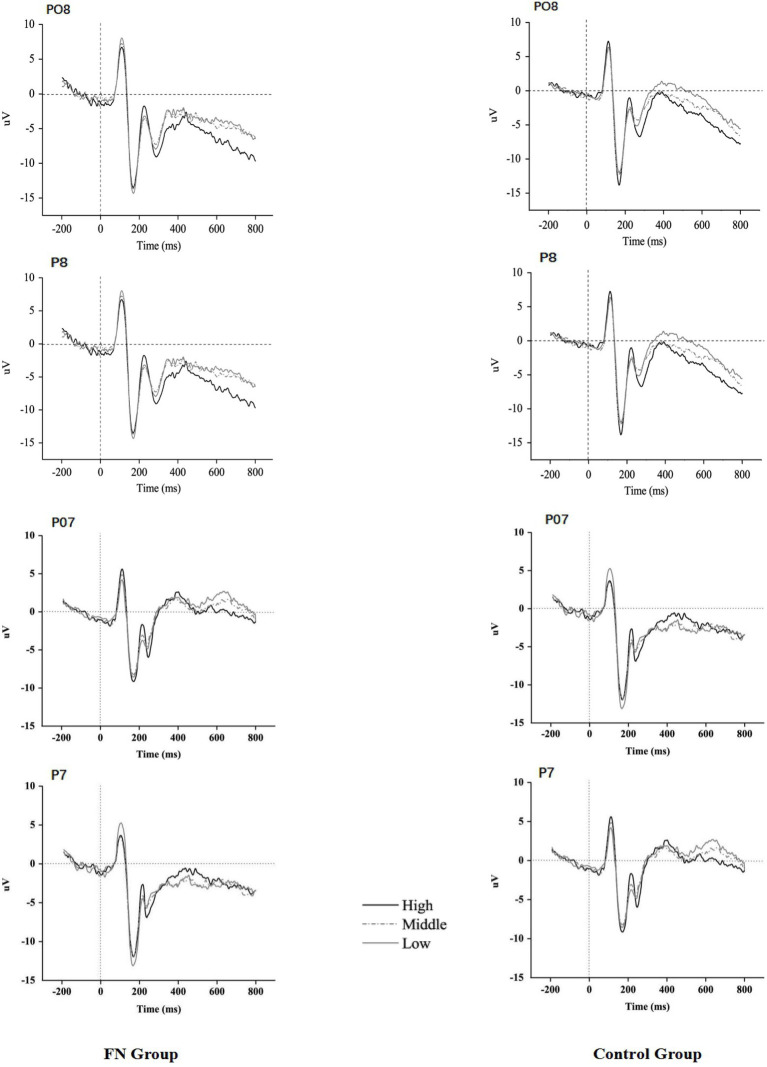
ERPs response recorded during the retrieval phase.

**Figure 6:**
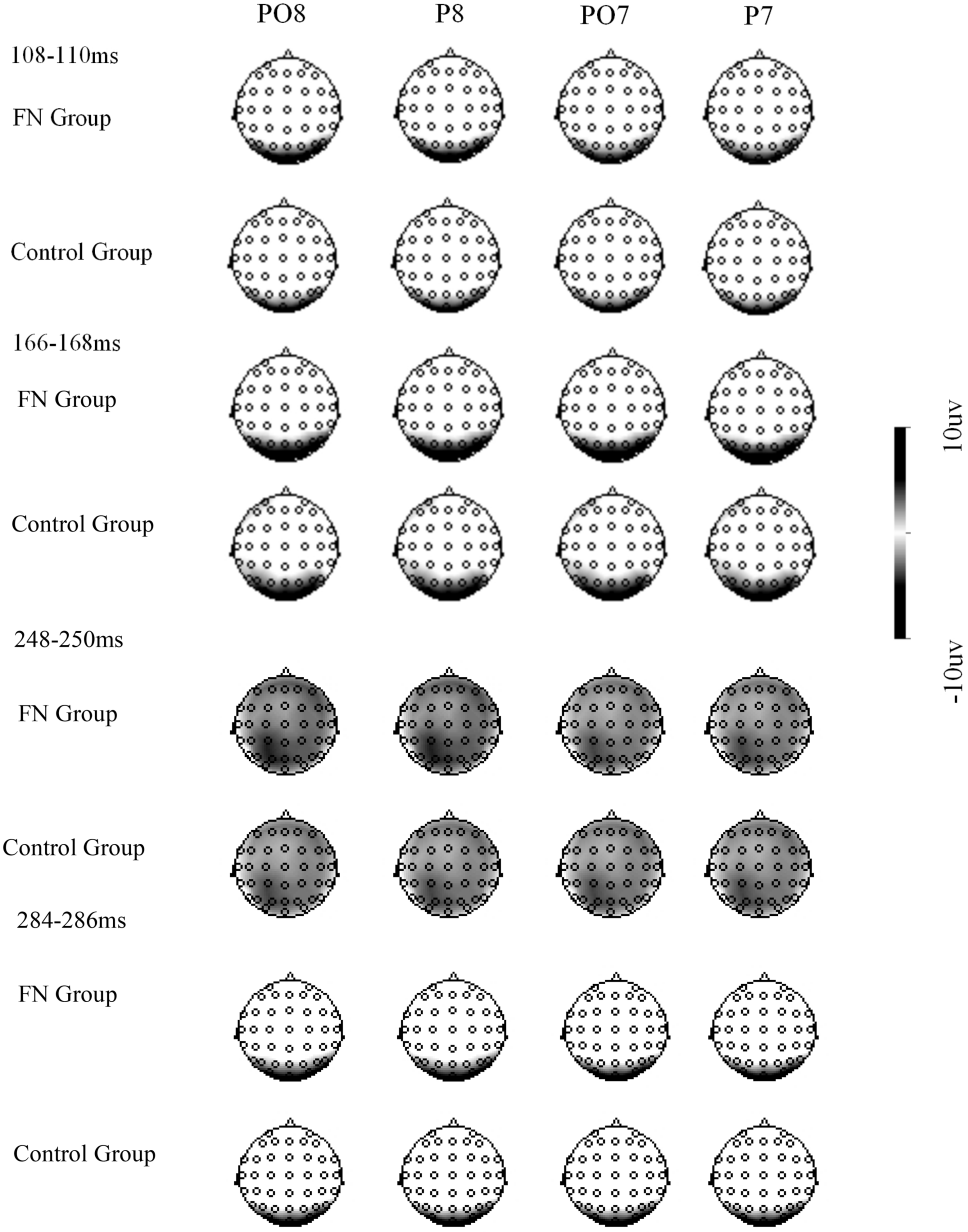
Differential topographical maps.

*P1* The 2 (Group: FN group/control group) × 3 (Picture type: HA, LA MA) × 2 (Electrode position: left and right) repeated measure ANOVAs were conducted on the peak and latency of P1. In the retrieving phase, the main effect for Group on the P1 latency period was significant (*p* = 0.047), and the FN group had a shorter P1 latency period relative to the control group. The interaction effects between Group and Picture type on the P1 peak in both the encoding and retrieval phases were significant (*F*(2, 58) =50.91, *p* = 0.001; *F*(2, 58) =3.87, *p* = 0.048). Simple effect analysis revealed that there was a significant difference in the FN group between the three types of pictures, and the P1 peak amplitude of the LA pictures was the largest (Ps < 0.05), with no significant difference between HA pictures and MA pictures (*p* = 0.285) In contrast, the control group had the highest P1 amplitude evoked by HA pictures in the learning phase, compared with MA pictures and LA pictures (*p* = 0.032, *p* = 0.034). However, there was no significant difference between MA pictures and LA pictures (*p* = 0.949). There was no significant difference in the task types’ impacts in the control group in the retrieving phase (Ps > 0.05). It indicated that the FN group exhibited early processing bias towards LA faces in both the encoding and retrieval phases, while the control group had early processing bias towards HA faces only in the learning phase. The other main effects and interaction effects were not significant (Ps > 0.05) (see Figures 3–6).

*N170* The 2 × 3 × 2 repeated measure ANOVAs were conducted on the peak and latency of N170. In the retrieving stage, the main effect of Group on the N170 latency period was significant (*F*(1, 29) =5.99, *p* = 0.028), and the latency period of N170 in the FN group was shorter than that in the control group. The interaction effects between Group and Picture type on the peak of N170 in both the learning and retrieving phases were significant (*p* = 0.001, *p* < 0.001). In the learning stage, the FN group had the largest peak of N170 for LA pictures, but there was no significant difference between HA pictures and MA pictures (*p* = 0.803). The pairwise comparisons between HA pictures, MA pictures, and LA pictures of this group were significantly different in the retrieving stage with *p* = 0.041, *p* = 0.066, and *p* < 0.001, respectively, in which LA pictures evoked the largest peak of N170, MA pictures the second, and HA pictures the lowest. In contrast, the control group in the learning phase had the largest N170 amplitude evoked by HA pictures, but there wasn’t a significant difference between MA pictures and LA pictures (*p* = 0.909). The pairwise comparisons between the FN, MA, and LA pictures in the retrieving stage were not significant. The other main effects and interaction effects were not significant (Ps > 0.05).

*P2* In the retrieving stage, the main effect of Picture type on the P2 latency period was significant (*F*(2, 58) =3.97, *p* = 0.045). The P2 latency period of the HA pictures was shorter than that of MA pictures and LA pictures (*p* = 0.037, *p* = 0.014), and the difference between MA pictures and LA pictures was not significant (*p* = 0.524). At the peak, the main effects of Group were significant in the two stages (*F*(1, 29) =5.12, *p* = 0.041; *F*(1, 29) =17.10, *p* = 0.001), and the P2 peak in the FN group was larger than that in the control group. The main effect of Picture type was significant (*F*(2, 58) =4.01, *p* = 0.046; *F*(2, 58) =11.19, *p* = 0.001), and HA pictures evoked the highest P2 amplitude, and LA pictures evoked the smallest P2 amplitude.

*N300* In the retrieving stage, the main effect of Picture type on the N300 latency period was significant (*F*(2, 58) =4.58, *p* = 0.031), and the latency period of the HA pictures was shorter than that of the LA pictures (*p* = 0.011). The main effect of Picture type on the N300 peak was also significant (*F*(2, 58) =39.08, *p* < 0.001), and the N300 amplitude evoked by HA pictures was significantly larger relative to MA pictures and LA pictures (Ps < 0.001), while there was no significant difference between MA pictures and LA pictures (*p* = 0.724). The other main effects and interaction effects were not significant (Ps > 0.05).

## Discussion

In experiments 1 and 2, the attentional biases of young females with facial dissatisfaction towards faces of different levels of attractiveness were examined. The results revealed that the FN group responded to the LA pictures significantly faster in the consistent condition than in the neutral condition, indicating that they showed an attentional orientation to the LA pictures. They reacted significantly faster to the LA pictures in the inconsistent condition than in the neutral condition, showing facilitation in attentional disengagement from LA pictures. For the HA pictures, the reaction times of the FN group in the consistent condition were significantly longer than in the neutral condition, showing that they exhibited attentional avoidance of the HA faces. The control group reacted more slowly to the HA pictures in the inconsistent condition than in the neutral condition, indicating that they had difficulty in attentional disengagement from the HA pictures. Therefore, regardless of whether faces are presented subliminally or supraliminally, females with facial dissatisfaction show attentional orientation to LA faces, facilitation in attentional disengagement from LA pictures, and attentional avoidance of HA faces. The control group exhibited difficulty in attentional disengagement from HA pictures.

As a sensitive stimulus, the LA face has an attention superiority effect, and females with negative face image will first direct their attention to LA faces. In this study, females with facial dissatisfaction show attentional orientation to LA pictures, which is consistent with the conclusions of previous research. Young females with facial dissatisfaction are more sensitive to negative face-related stimuli, resulting in faster detection of negative face-related stimuli. But then they avoided LA pictures manifested as facilitation in attentional disengagement from LA faces, partly inconsistent with previous studies. For example, in a study on attentional bias toward body words, females with obesity were found to be vigilant about body words but not attentional avoidance (von Spreckelsen et al., 2018). The reason may be the low arousal intensity of body words and the simplicity of the task, which required few cognitive processing resources. However, this study used highly stimulating pictures that can fully activate negative body images and further attentional avoidance. The attentional “vigilance-avoidance” model in the study of anxiety disorders would function as evidence for the above conclusion(Caldwell and Newman, 2005; Mitchison and Mond, 2015; Jacoby et al., 2016). The model supposes that anxious individuals will notice threatening stimuli sooner at first and then disengage to avoid further detailed processing(Koster et al., 2004). This information processing mode will maintain anxiety because anxious individuals identify threat information faster. However, without precise processing, the participants failed to evaluate the authenticity and objective threats of the information. Consequently, their estimation of threats comes from self-overestimation. Therefore, their anxiety was maintained. Additionally, avoiding facts cannot ease but maintain this state. Accordingly, females with facial dissatisfaction may also have such a vigilance-avoidance cognitive mechanism. They detect low-attractive face information faster and then avoid it and further detailed processing. Additionally, they also avoid HA pictures. This processing mode maintains the anxiety state of facial dissatisfaction about appearance and prevents the conduction of objective evaluation toward stimuli. This vigilance-avoidance cognitive mechanism may also be one of the reasons why females have negative emotional experiences and then conduct or maintain problem acts.

Participants in the control group only showed the difficulty of attentional disengagement from HA pictures, which was consistent with the conclusions of previous studies and reflected their beauty bias. Some studies have found that as a reward (Rhodes, 2006; Marzi and Viggiano, 2010), beautiful faces contain greater reward value (Ishai, 2007; Sui and Liu, 2009; Halford and Hsu, 2020) and are favored by individuals (Luo, 2013; Ma-Kellams et al., 2017). In this study, it may be that the greater reward value triggered longer glance and slowed down the reaction time.

Experiments 3 used the learning-retrieving test paradigm to compare the differences in learning-retrieving behaviors and ERPs between the two groups on HA, MA, and LA pictures. Behavioral data show that, compared to MA pictures, only the FN group completed judging LA pictures in a shorter reaction time and at a higher retrieving rate, while there was a longer reaction time and lower retrieving rate for HA pictures. There were no significant differences in the response time or retrieval rate among the three types of pictures in the control group. This result is consistent with our hypothesis, which aligns with the influence of negative body images on the cognitive processing of information. The processing of body information impacted negative body images. When information related to the negative schema (such as weight, body shape, and food information) appears in the presentation or environment, the negative schema will be activated, thereby facilitating the processing of schema-consistent information, inhibiting or contradicting the processing of schema-inconsistent information(Levine and Piran, 2004; Chen and Jackson, 2005). Consequently, the participants pay attention to, judge, and store information selectively in different information processing stages (Chen and Jackson, 2006; Cho and Lee, 2013), which is similar to the fat negative schema conclusion. Females with facial dissatisfaction rate their faces as lowly attractive and selectively process LA pictures that are consistent with their schema, which enables them to process LA pictures faster and more intensively, so the retrieving rate is better. In contrast, their contradiction to schema-inconsistent information on HA pictures contributes to a lower rate and a slower speed of retrieval.

The ERP results showed that during the learning phase, the LA pictures induced the highest P1 and N170 amplitudes in the FN group, and HA pictures were the lowest. While the control group was the opposite, participants had a preference for HA pictures, and HA pictures induced the highest P1 and N170 amplitudes. In the retrieving phase, the LA pictures of the FN group induced the highest P1 and N170 amplitudes, and the HA pictures caused comparatively lower amplitudes of P1 and N170, but there was no such difference in the control group. This shows that participants in the FN group exhibited a preference for LA pictures in both the learning and retrieving stages. They learned and remembered LA pictures better, while neutral participants preferred HA pictures.

P1 is considered to be the reflection of the early stage of face processing, the rough facial representation and the retrieval of the simple facial features (Itier and Taylor, 2004; Kranz and Ishai, 2006). A study employed real and virtual faces to compare the processing differences between sad and neutral facial expressions and found that sad expressions cause stronger P1 than neutral expressions within a time window of 80 ~ 110 ms (Kramer et al., 2020). In attention-oriented research, P1 is often considered to be related to early attention and low-level physical classification of stimuli (Itier et al., 2007). Early studies believed that only physical features of complex visual stimuli were processed in low-level brain regions within 100 ms after presentation (Pizzagalli et al., 2002), while specific processing occurred at the later stages. However, recent studies on psychophysiology and electrophysiology have shown that visual stimulus classification and processing may begin approximately 100 ms after the stimulus is presented (Thorpe et al., 1996; Kranz and Ishai, 2006). For example, in the experiment of Thorpe et al., the participants were required to determine whether a series of pictures contained animal pictures swiftly and found swift visual classification may occur 100–150 ms after the stimulus was presented(Thorpe et al., 1996). Liu and his team captured a greater P1 amplitude induced by faces than that induced by other image stimuli, which is supposed to prove a greater involvement of attention resources in face processing (Sui and Liu, 2009) (see Table 2).

**Table 2:** ERPs amplitude in study phase (*M* ± *SD*).

Type	P1(uV)		N170(uV)		P2(uV)		N300(uV)	
	FN group	Control group	FN group	Control group	FN group	Control group	FN group	Control group
High	6.64 ± 0.75	8.16 ± 1.16	−10.40 ± 1.11	−12.604 ± 1.03	4.50 ± 0.99	1.13 ± 1.02	−8.83 ± 0.63	−5.90 ± 0.75
Middle	6.31 ± 0.80	6.66 ± 1.19	−10.20 ± 1.01	−12.511 ± 0.97	3.82 ± 0.72	0.64 ± 0.87	−7.52 ± 0.62	−4.37 ± 1.00
Low	7.83 ± 0.80	6.64 ± 1.19	−12.08 ± 1.00	−10.571 ± 1.04	3.53 ± 0.95	0.03 ± 0.96	−6.91 ± 0.79	−4.14 ± 0.95
Total					3.98 ± 0.87	0.56 ± 0.94	−7.75 ± 0.66	−4.81 ± 0.87

N170 is a unique component in face processing that is usually captured in the temporal-occipital region, representing the retrieval of face processing and the spatial relationship between facial parts (Hinojosa et al., 2015). The study of facial feature coding found that facial features affect the latency period and amplitude of the N170 (Eimer and Williams, 2000). Faces with expressions (sadness, fear, disgust, anger, surprise, happiness) induced a shorter latency period and greater N170 than neutral expressions. The N170 latency period induced by positive expressions was shorter than that induced by negative expressions, and the N170 amplitudes induced by fear expressions were greater than those induced by neutral and surprised expressions (Hinojosa et al., 2015). The amplitude of the N170 component is also affected by the attractiveness of the face. Some studies present participants with high, medium, low, and unattractive faces, and it is found that compared with medium and low-attractive faces, the N170 latency period induced by high-attractive faces is shorter and the amplitude is greater (Joyce and Rossion, 2005; Tanaka, 2018). A new study also found that compared to medium and unattractive faces, the N170 component of both genders posed an electrophysiological bias toward attractive faces (Tanaka, 2018). The more attractive individuals are, the smaller the electrophysiological bias toward unattractive faces in the environment, indicating a smaller involvement of attentional resources.

The components of P1 and N170 reflect the situation of the early processing stage of faces. At the early stage, the FN group has a cognitive bias toward LA pictures. The ERP effect induced by LA pictures in learning and retrieving tasks is the strongest, and that induced by HA pictures is the weakest. This reflects the information processing features of schema participants from the perspective of brain mechanisms. Young females with facial dissatisfaction believe that their faces are less attractive, and LA pictures as schema-consistent information involve more cognitive processing resources and a greater degree of processing. On the other hand, they cognitively reject HA pictures, which leads to less involvement of face processing resources. Therefore, FN will show a cognitive bias toward different facial stimuli in the early stage. They invest more resources in LA pictures that are consistent with the negative schema, resulting in greater processing intensity and better memorization. This feature is similar to the result of Smith’s research on persons with body image disorders. They found that individuals with body disorder pay more attention to unsatisfactory body parts, while normal participants pay more attention to satisfying parts. The degree of attention of individuals with body image disorder to unsatisfactory parts is related to their subsequent dietary regulation acts (Massar et al., 2011; Gupta et al., 2019). In addition, some attentional bias studies on anxiety schema and diet disorder participants found that schematists rapidly avoid threatening information, which is manifested by individuals’ attentional avoidance of threatening information. To avoid information refinement to reduce or combat the anxiety or negative emotional experience that threatening information may induce. This avoidance strategy prevents individuals from objectively evaluating the threat of stimuli, which leads to the continuation of the original state (Boon et al., 2000; Rinck and Becker, 2006; Vrijsen et al., 2009; Hollitt et al., 2010; Wilson and Wallis, 2013). Therefore, those with a negative appearance schema overprocess LA pictures in the environment and avoid HA pictures as threatening information. Such cognitive overprocessing of the consistent information of the negative schema may lead to the overestimation of information threat. Their resistance to inconsistent information and avoidance strategy lead to their insufficient awareness of objective information and exacerbate their dissatisfaction with their appearance, which further enhances the schema so that the negative schema is consolidated and maintained, which in turn leads to the continuation of some problematic behaviors.

Participants in the control group exhibited a preference for HA pictures in the judgment task, which is consistent with previous research results on attractive faces and may reflect that the activation of the reward value of beautiful faces is more popular (Atari et al., 2017; Ma-Kellams et al., 2017; Arnocky, 2018). In addition to analyzing individuals’ preference for beautiful faces from the perspective of internal reward value, we can also explain it from the perspectives of evolutionary psychology and cognitive processing psychology. Evolution-oriented theories believe that facial beauty is related to better environmental adaptation and evolution. This better adaptation and evolutionary ability have also been defined as the reasons for a better reproduction success rate and good genes in the long evolutionary history of mankind (Shackelford and Larsen, 1999; Henderson and Anglin, 2003). According to the good genes theory, the higher the attractiveness of an individual’s face, the stronger the individual’s body immunity, the healthier the genes, and the better the reproductive ability. In addition, those individuals with lower attractiveness leave us with the a similar impression as bad food, making us feel unhealthy and immature. Individuals with low attractiveness also seem to be associated with poor health, low survival rates, and a threat to the continuity of race, eventually being disfavored by others. Cognitive orientation suggests that the preference for attractive faces may merely be the preference for familiar stimuli.

During the retrieving stage, participants in the control group exhibited no difference in retrieving the three types of pictures, indicating that they did not pose a memory preference for attractive faces. Some studies have reported differences in retrieving attractive faces (Eimer and Williams, 2000; Joyce and Rossion, 2005; Liu et al., 2016), which may come from the mixed participants of both genders. Such research mostly arranges mixed male and female participants and applies stimulates faces of mixed genders. Studies have shown that for observers of different genders, faces have different internal reward values (Joyce and Rossion, 2005; Tanaka, 2018), and gender differences can cause differences in face processing and memory. Therefore, compared with the settings of single-sex participants and face pictures in this study, mixed-sex experiments will be interfered with by more irrelevant variables, which affects the early components in the retrieving phase. This study selected only female participants and facial stimuli, thereby controlling the influence of irrelevant variables to minimize the experimental error.

The P2 component amplitude is attributed to the main effect of grouping and task type. The FN group has a higher amplitude than the control group, the high attractiveness images draw the highest amplitude, and the low-attractiveness images draw the smallest amplitude. The N300 amplitude is attributed to the main effect of task type, and HA pictures induces a significantly larger amplitude than MA pictures and LA pictures. P2 reflects the early processing of visual stimuli by the brain and is a sign of perceptual processing (Ralph-Nearman et al., 2019; Carlson, 2021). Some studies have found that the amplitude of P2 is related to attention involvement (Frenkel and Bar-Haim, 2011; Kanske et al., 2011). The larger the amplitude, the more attention involvement there is (Massar et al., 2011; Rossignol et al., 2012; Schindler and Bublatzky, 2020), indicating that once entering the perceptual processing stage, the two groups of participants have the same cognitive bias toward faces, and both groups are more involved in HA pictures. We speculate that this reflects the difference in the processing mechanism affected by the schema.

Specifically, for those with a negative appearance schema, reports have pointed out that individuals with a negative schema also have an implicit self-esteem effect Chen and Jackson, 2006; Chen et al., 2007). Research on individuals with depressive schema has found that their cognitive basis consists of disavowal and negative schema (Chen and Jackson, 2005; Verplanken and Velsvik, 2008; Pawijit et al., 2019), such as encoding schema-related neutral stimuli as negative stimuli. As a result, individuals with a negative appearance schema are dissatisfied with their looks and suppose their faces are low-attractive. Their cognitive basis is the identification of LA pictures. HA pictures are contrary to this and are regarded as threatening stimuli to be processed. In other words, when entering the perceptual stage, individuals with a negative appearance schema process HA pic as threat stimuli. From the perspective of evolutionary psychology, compared to neutral information, stimuli containing emotions can indeed be highlighted in the environment and can attract individual attentional resources (Ohman et al., 2001; Batty and Taylor, 2003; Massar et al., 2011; Veenstra and de Jong, 2012). In the process of biological evolution and adaptation, negative stimuli, especially those containing threatening or survival-related information, attract more attention. Putting more attentional resources on threatening information manifests biological adaptability as well. Individuals with a negative self-image may regard HA pictures as a kind of threatening information contrary to their schema. After entering the perception stage, HA pictures, as threatening information, can quickly attract more processing resources and greater attentional involvement. Therefore, the ERP effect is greater.

The activation of the right occipital lobe of those with negative physical self-image in the retrieving stage found on the EEG may represent the process of self-information processing (Levine and Piran, 2004; Morita et al., 2008; Pitcher et al., 2011). This likely indicates that the activation of the right occipital lobe is related to the processing of self-image information, which is consistent with the results of this study. This may also indicate that the brain area is related to a negative body schema.

## Limitations

Pictures were the only stimuli used in the current study, which cannot comprehensively reflect the cognitive processing approach of females with facial dissatisfaction toward different stimuli. Further research would involve word and sound stimuli to explore the information processing features of females with facial dissatisfaction. This research activated the negative body image of appearance from the perspective of explicitness (attractiveness judgment) and discovered the processing and memory preferences of the negative body image among participants, but it is still unclear whether this preference exists when body information is implicitly activated. Therefore, further research can also focus on exploring how preference is expressed when body information is implicitly activated.

## Conclusion

1. Young females with facial dissatisfaction exhibit attentional orientation and attentional avoidance toward LA pictures and attentional avoidance toward HA pictures.
2. Young females with facial dissatisfaction identify and retrieve LA pictures in a faster and more accurate way.
3. The P1, N170, P2, and N300 amplitudes induced by LA pictures are higher, reflecting the difference in the processing mechanism.
4. Young females with facial dissatisfaction tend to pay attention and conduct memory processing when encountering LA pictures.

## Data availability statement

The raw data supporting the conclusions of this article will be made available by the authors, without undue reservation.

## Ethics statement

Subjects gave written, informed consent, and our procedures and protocols were approved by the Academic Committee of School of Education and Psychology, Southwest Minzu University (Experiment number: 20200911–023). Written informed consent was obtained from the individual(s) for the publication of any potentially identifiable images or data included in this article.

## Author contributions

LZ, HZ, XW, XM, and QL contributed equally to the conception of the idea, implemented and analyzed the experimental results, wrote the manuscript, and read and approved the final manuscript. All authors contributed to the article and approved the submitted version.

## Funding

This study was supported by Fund for Chinese Central Universities (Grant number: skqy201212) and the Centre for Applied Psychological Research of the Education Department of Sichuan Province (Grant number: CSXL-182004).

## Conflict of interest

The authors declare that the research was conducted in the absence of any commercial or financial relationships that could be construed as a potential conflict of interest.

## Publisher’s note

All claims expressed in this article are solely those of the authors and do not necessarily represent those of their affiliated organizations, or those of the publisher, the editors and the reviewers. Any product that may be evaluated in this article, or claim that may be made by its manufacturer, is not guaranteed or endorsed by the publisher.
